# Mexican case report of a never‐treated Laron syndrome patient evolving to metabolic syndrome, type 2 diabetes, and stroke

**DOI:** 10.1002/ccr3.1193

**Published:** 2017-09-27

**Authors:** Inma Castilla‐Cortazar, Giovana Femat‐Roldán, Joel Rodríguez‐Rivera, Gabriel A. Aguirre, Mariano García‐Magariño, Irene Martín‐Estal, Luis Espinosa, Carlos Díaz‐Olachea

**Affiliations:** ^1^ Escuela de Medicina Tecnologico de Monterrey Monterrey México; ^2^ Fundación de Investigación HM Hospitales Madrid Spain; ^3^ Neurocenter Monterrey Nuevo León México

**Keywords:** GH insensitivity, GH/IGF‐1 axis, GHR, IGF‐1, Laron syndrome, Laron syndrome, metabolic syndrome, obesity, stroke, type 2 diabetes

## Abstract

Glucose and lipid profile together with blood pressure should always be considered for low sera‐IGF‐1 patients. Even when adulthood is reached, IGF‐1 therapy in these patients should be pursued as metabolic and protective cellular effects could be triggered. Real incidence of growth hormone insensitivity is still to be uncovered.

## Introduction

Laron syndrome (LS) or growth hormone insensitivity (GHI) is a condition given by normal‐high basal GH and low IGF‐1 presented with proportionate dwarfism 1. The metabolic impact of IGF‐1 chronic deprivation – starting *in uterus* – affects directly or/and indirectly to carbohydrate and lipid metabolism. One of the first symptoms is hypoglycemia 2, 3, 4, 5. Obesity, another common symptom, is related with compensatory mechanisms aiming normalization of hypoglycemia (sex hormones, glucocorticoids, among others) 5, 6. Of great interest, the study of Laron's disease attains to the dynamic changes observed in carbohydrate and lipid metabolism among the older never‐treated patients with LS.

At first, during early childhood, these patients present hypoglycemia. Later on, they progressively develop hyperinsulinemia followed by hypoinsulinemia, glucose intolerance and, around puberty, even diabetes and obesity with hyperlipidemia (especially cholesterol and triglycerides). Thus, untreated patients with LS develop metabolic syndrome (MetS) and type 2 diabetes (T2D) 7.

As different opinions still persist concerning these metabolic disorders 8 in never‐treated patients with LS; herein, we contribute by presenting the case of a never‐treated GHI patient with a dramatic ending.

## Case Presentation

We present the case of a 53‐year‐old female Mexican patient, introduced to our group after *de visu* identification, short stature, high‐pitched voice, and obesity. She developed obesity at a young age and was later diagnosed with metabolic syndrome, and type 2 diabetes later. Several stroke events due to hypertensive crisis recently occurred, leading to her decease.

The patient developed obesity in her puberty and was diagnosed with type 2 diabetes and arterial hypertension at the age of 43 after noticing unrecorded weight loss as well as hyperglycemia symptomatology. She also suffered from bilateral cataracts that required surgery.

The patient had poor adherence to the medical treatment using captopril 50 mg once a day and decided to stop the 30 daily units of subcutaneous NPH insulin she used 6 months prior to the interrogation. In addition, she never used medication to treat her recently diagnosed hyperlipidemia.

## Physical Examination

Her initial physical examination when accepted for short stature evaluation revealed an hypoplastic nasal bridge, high‐pitched voice, visceral fat, hip dysplasia, and *genu valgum*. Her body mass index was of 31.55 kg/m^2^ with a weight of 71 kg and a height of 1.50 m. The measurement of her waist was of 76.4 cm, 42.4% of body fat, and visceral fat area of 104.3 cm^2^. It should be noted that the patient began suffering from gradual weight loss since her diagnosis. Before, her body mass index was of 39.55 kg/m^2^. Vital signs showed a heart rate of 88 beatings per minute and a blood pressure of 160/88 mmHg.

Blood tests were performed to reveal her metabolic state (Table 1): high fasting glucose, triglycerides, total cholesterol, LDL, and low HDL. The GH/IGF‐1 axis presented altered values. IGF‐1 was half the expected with very low GH basal levels and no response to clonidine stimulation, a common finding in never‐treated adult patients with Laron Syndrome. This fact is possibly owing to pituitary exhaustion. Whole exome sequencing revealed several polymorphisms within GHR postreceptor molecules. However, due to the patient's decease, no genetic and functional confirmation could be achieved.

**Table 1:** Serum analytical parameters, serum circulating IGF‐1 levels, and GH‐stimulation test

Parameter	Serum value	Reference range
Glucose	**239 mg/dL**	60–100 mg/dL
Cholesterol	**220.0 mg/dL**	<200 mg/dL
Triglycerides	**316 mg/dL**	<150 mg/dL
HDL Cholesterol	**32.0 mg/dL**	60 mg/dL
LDL Cholesterol	124.8 mg/dL	60–130 mg/dL
IGF‐1	**41.60 ng/mL**	87–238 ng/mL
Growth Hormone (Clonidine Stimulation)		
Basal (0 min)	**<0.05 ng/mL**	>8 ng/mL
30 min	**<0.05 ng/mL**	>13 ng/mL
60 min	**0.18 ng/mL**	>13 ng/mL
90 min	**1.30 ng/mL**	>13 ng/mL
120 min	**0.25 ng/mL**	>13 ng/mL

HDL, high‐density lipoprotein; LDL, low‐density lipoprotein, IGF‐1, insulin‐like growth factor. Highlighted values are those out of reference range.

**Figure 1:**
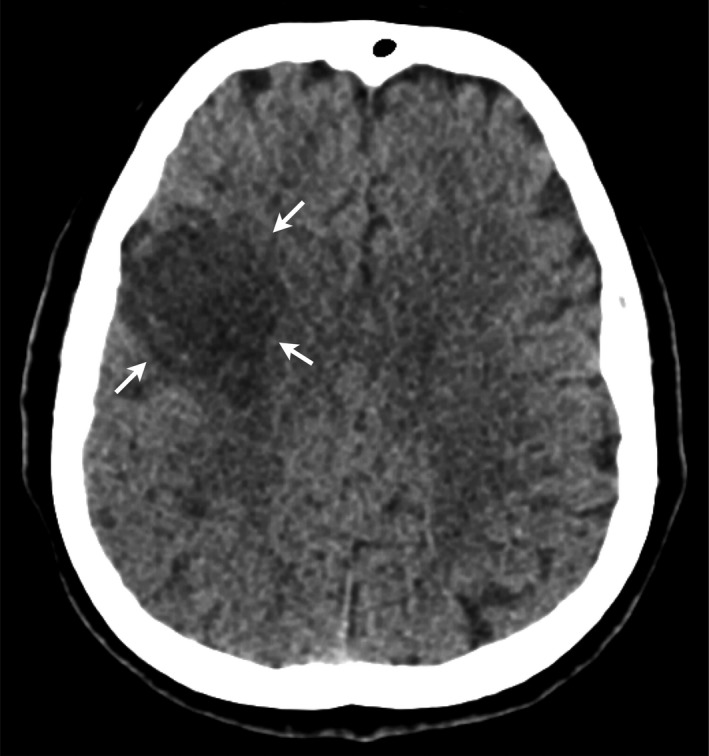
Axial noncontrasted computed tomography demonstrates a diffuse hypodense area in the inferior frontal and precentral gyrus in the middle cerebral artery territory.

Four days after initial evaluation, the patient manifested acute neurological deficit characterized by left hemiparesis and dysarthria, yet did not seek medical attention until a week later. Vital signs yielded a blood pressure of 190/90 mmHg and a heart rate of 100 bpm. Computed tomography showed a right frontal hypodense area involving the right frontal lobe above the Sylvian fissure consistent with infarction in the superior division of the right middle cerebral artery (Fig. 1). She then received medical treatment.

Physical examination a week after her first stroke showed left hand paresis and minor left facial paralysis, with a National Institute of Health Stroke Scale of 2. A large vessel disease was pointed as responsible for the strokes. A carotids and heart image analysis were prescribed as to elucidate the etiology of the condition. Neurological follow‐up was not performed as the patient missed subsequent appointments. Months later, after new hypertensive crisis and other cerebrovascular events, she was admitted into an intensive care unit where she perished.

## Discussion

In 1966, Laron et al. 9 described an alteration that presented severe growth retardation and obesity as main physical signs. Such alteration presented with normal‐high basal GH and low IGF‐1 1 contrary to pituitary dwarfism (or Growth Hormone Deficiency – GHD) which presented both IGF‐1 and GH low serum levels. Both their phenotypes are *de visu* clearly evident. Patients with GHD present low stature, no longitudinal bone growth but normal muscle development, hence appearing as disproportionate limbs and head 10. By contrast, Laron dwarfism courses with hip dysplasia (commonly), dental, eye and hearing problems, low stature due to longitudinal bone growth fail, but underdeveloped musculature, hence giving the impression to be proportionate: like children 1, 10, 11. Moreover, *fascia* features of both phenotypes are very characteristic, being extensively reviewed elsewhere 1, 10, 11. patients with Laron syndrome also developed several metabolic conditions, such as severe hypoglycemia in the early childhood, caused by a low glucose output from the liver in the absence of IGF‐1 5. Never‐treated adult patients also presented hyperinsulinemia that progresses to hypoinsulinemia and glucose intolerance, with the installment of type 2 diabetes and obesity with high cholesterol and low‐density lipoproteins from puberty onward 5, 12. It has been elucidated that the natural evolution of IGF‐1 deprivation leads to the development of Metabolic Syndrome due to metabolic deregulation, where IGF‐1 plays a pivotal role 1, 13. Interestingly, a recent study found an altered genetic expression pattern for proteins implicated in carbohydrate and lipid metabolism in mice with partial IGF‐1 deficiency 14.

Also, IGF‐1 possesses cardio‐protective and antiaging effects, where low circulating levels of this hormone contribute to the development of a cardiac aging phenotype 15. Of interest, a relationship between IGF‐1 and several cardiovascular risk markers has been found, some of them being arterial hypertension, diabetes, endothelial dysfunction, and carotid intima‐media thickness 16. Moreover, IGF‐1 has been demonstrated to play a pivotal role in the development of the brain and bony structures of the cranium 12.

In the present work, we report the case of a patient with Laron Syndrome and describe the progression to metabolic syndrome, type 2 diabetes, and obesity with a dramatic outcome. Realistic Laron Syndrome prevalence is yet unknown, as the majority of cases remain undiagnosed around the world. This is the case in Mexico, where our group has found patients with *de visu* diagnosis. Some of them are still being confirmed, while others have been published 17.

In conclusion, patients with IGF‐1 deficiency tend to be misdiagnosed with pituitary dwarfism. In addition, the unaffordability of substitutive therapy results in many cases remaining undiagnosed and untreated. As this work reveals, not only prepubescent individuals suffering from this condition should be medicated with IGF‐1, but also adults could be provided with it to avoid dramatic complications derived from the long progression of metabolic syndrome.

## Informed Consent

A copy of the patient's informed consent is available via the corresponding author.

## Conflict of Interest

The authors declare there is no conflict of interest.

## Authorship

ICC: involved in patient referral, clinical diagnosis, molecular diagnosis design, and reviewed the manuscript; GFR: participated in neurological procedures following stroke and wrote the manuscript; JRR: contributed to clinical management and wrote the manuscript; GAA: contributed to the processing of samples, stablished molecular diagnosis, reviewed the literature, and wrote the manuscript; MGM: contributed in clinical management and sample extraction; IME: processed the sample and reviewed the literature; LE: involved in neurological procedures; and lastly, CDO: participated in clinical analysis and profiling.
